# Low‐Grade Serous Carcinoma in Association With Endosalpingiosis in a Cervical Lymph Node: A Case Report and Literature Review

**DOI:** 10.1002/dc.70206

**Published:** 2026-09-01

**Authors:** Nyein Nyein Htun, Nour Sneige

**Affiliations:** ^1^ Department of Pathology The University of Texas MD Anderson Cancer Center Houston Texas USA

**Keywords:** cervical lymph nodes, ectopic Müllerian tissue, endosalpingiosis, low‐grade serous carcinomas, Müllerian inclusions, serous borderline tumors

## Abstract

Endosalpingiosis is a benign Müllerian inclusion that rarely involves supradiaphragmatic lymph nodes. Malignant transformation in the setting of nodal endosalpingiosis is exceptional. We report a case of low‐grade serous carcinoma arising in a cervical lymph node in the setting of endosalpingiosis in a 41‐year‐old woman presenting with Horner syndrome. Imaging revealed a densely calcified, hypermetabolic supraclavicular lymph node. Fine needle aspiration and core biopsy demonstrated tubal‐type epithelium consistent with endosalpingiosis adjacent to a neoplastic micropapillary and cribriform proliferation with psammoma bodies. Immunohistochemistry showed positive staining for PAX‐8, estrogen receptor, and WT1, supporting Müllerian origin. The patient had a remote history of serous borderline tumor with noninvasive implants and *KRAS* and *MAPK1* mutations. No recurrent pelvic disease was identified on positron emission tomography–computed tomography. Neck dissection confirmed low‐grade serous carcinoma arising in association with nodal endosalpingiosis. This rare presentation underscores the importance of recognizing Müllerian proliferations in cervical nodes to avoid misdiagnosis as metastatic carcinoma.

## 1 Introduction

Endosalpingiosis is a benign Müllerian inclusion that consists of glands and cystic spaces lined by tubal epithelium. The inclusions are most commonly found in the female pelvis, including the uterine serosa, fallopian tube serosa, ovaries, round and uterosacral ligaments, cul‐de‐sac, and bladder. These inclusions have also been reported in the peritoneum, appendix, lymph nodes, and omentum [1]. Most cases of nodal endosalpingiosis are subdiaphragmatic; supradiaphragmatic nodal endosalpingiosis is exceedingly rare, and most of the reported cases involved axillary nodes [2, 3], or rarely, cervical nodes [4]. This nodal involvement by inclusions, especially in the absence of a history of a primary serous borderline tumor (SBT) or low‐grade serous carcinoma (LGSC), has resulted in mistaken diagnoses of metastatic breast carcinoma and metastatic papillary thyroid carcinoma. Distinguishing supradiaphragmatic nodal endosalpingiosis from metastatic carcinoma, in particular metastatic breast carcinoma in cases of axillary nodal endosalpingiosis or papillary thyroid carcinoma in cases of cervical nodal endosalpingiosis, is essential for appropriate patient management. Here, we report a case of a cervical lymph node with LGSC in association with nodal endosalpingiosis in a patient with a distant history of SBT without identifiable recurrent tumors in the surgical bed.

## 2 Case Presentation

A 41‐year‐old woman presented to the local emergency room with neuro‐ophthalmological symptoms. Her symptoms included pain in the left side of the neck, swelling and redness of the left eye, and drooping of the left eyelid, consistent with Horner syndrome. The patient also reported that her left pupil was smaller than her right pupil. Computerized tomography (CT) demonstrated a diffusely and densely calcified approximately 2.3 × 1.5 × 2.5 cm lobulated mass in the left side of the neck at the level of the thyroid gland, lateral to the common carotid artery and anterior to the proximal left vertebral artery, without invasion into adjacent soft tissues or vasculature. A positron emission tomography–CT (PET/CT) scan demonstrated an irregular hypermetabolic calcified left supraclavicular node with a standardized uptake value of 8.9, consistent with malignancy.

Given the Horner syndrome signs and symptoms, cervical lymph node fine needle aspiration and core needle biopsy were performed.

The cytologic preparation (liquid‐based) from fine needle aspiration showed a lymphoid tissue component and papillary and 3‐dimensional clusters of uniform, small to medium‐sized epithelial cells with minimal nuclear atypia (Figure 1). The core needle biopsy specimen showed 2 distinct components: ciliated columnar epithelium forming a small glandular structure with intercalated peg cells, consistent with endosalpingiosis (Figure 2), and a neoplastic epithelial cell population with small nests and micropapillary architecture, as well as free‐floating individual epithelial cells within unlined clear space and scattered psammoma bodies. Nuclear atypia was mild. The overall features were consistent with papillary LGSC (Figure 2).

**FIGURE 1:**
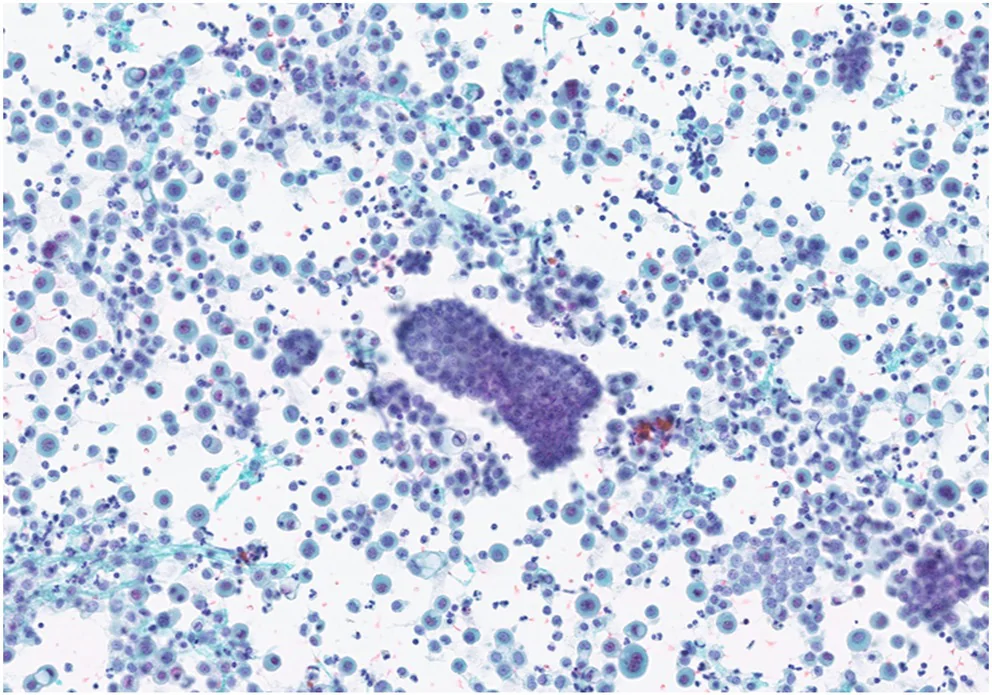
Fine needle aspirate from left supraclavicular lymph node (liquid‐based preparation) showing papillary structures with mild epithelial atypia, consistent with involvement by a low‐grade serous neoplasm.

**FIGURE 2:**
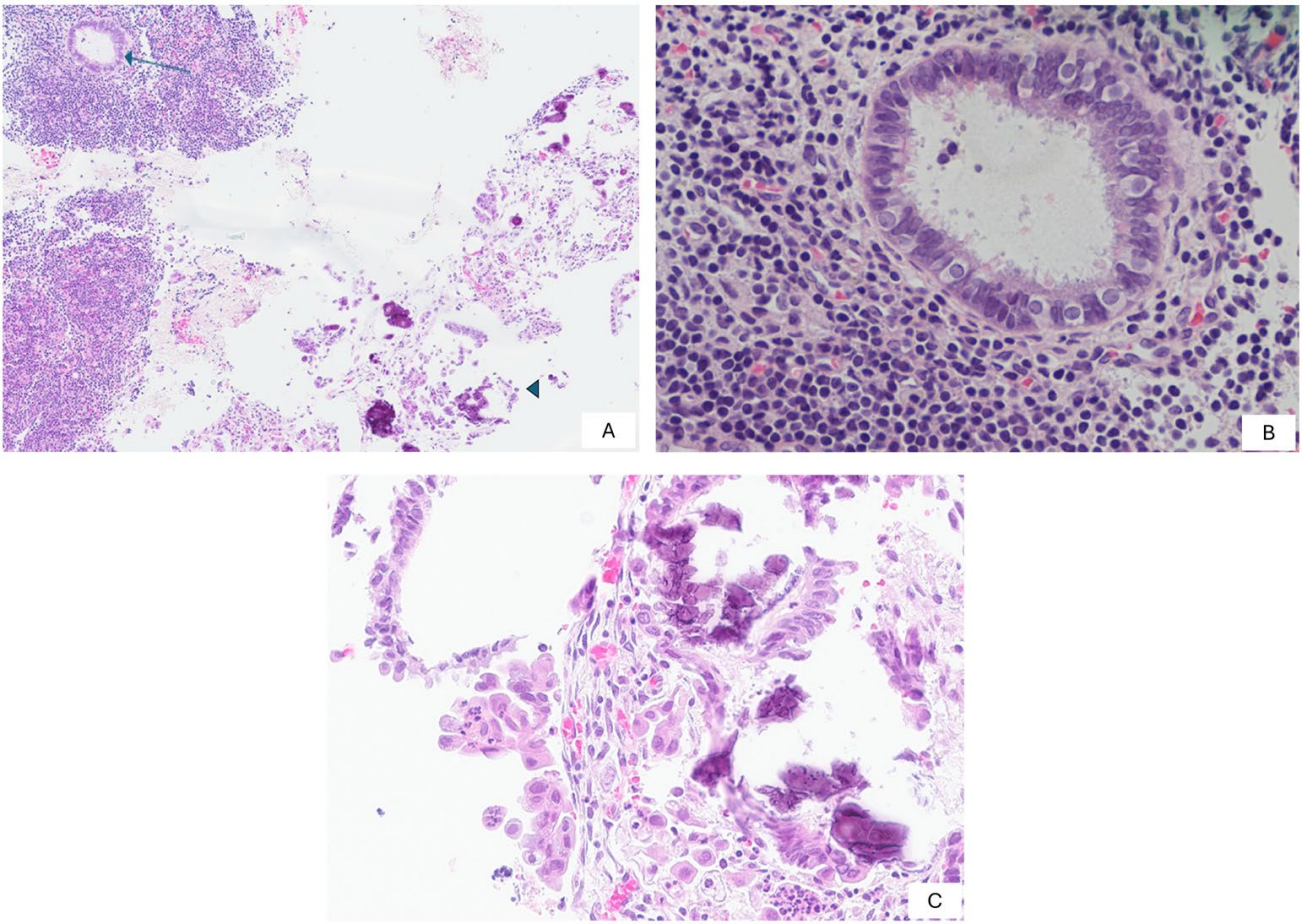
Tissue section of core needle biopsy specimen from left supraclavicular lymph node. (A) A low power view showing a cystic structure lined by a single layer of epithelial cells (arrow), characteristic of endosalpingiosis, and a few scattered small papillary structures and infiltrating individual epithelial cells with psammomatous calcifications (arrowhead), consistent with low‐grade serous neoplasm. (B and C) High‐power magnification views showing endosalpingiosis (B), and small nests and papillae as well as free‐floating individual epithelial cells within unlined clear space consistent with LGSC (C).

On immunohistochemistry, core needle biopsy specimens of cervical lymph node were positive for PAX‐8, estrogen receptor, and WT1 (Figure 3). The immunophenotype and cytomorphology supported Müllerian‐origin inclusion and low‐grade serous tumor differentiation.

**FIGURE 3:**
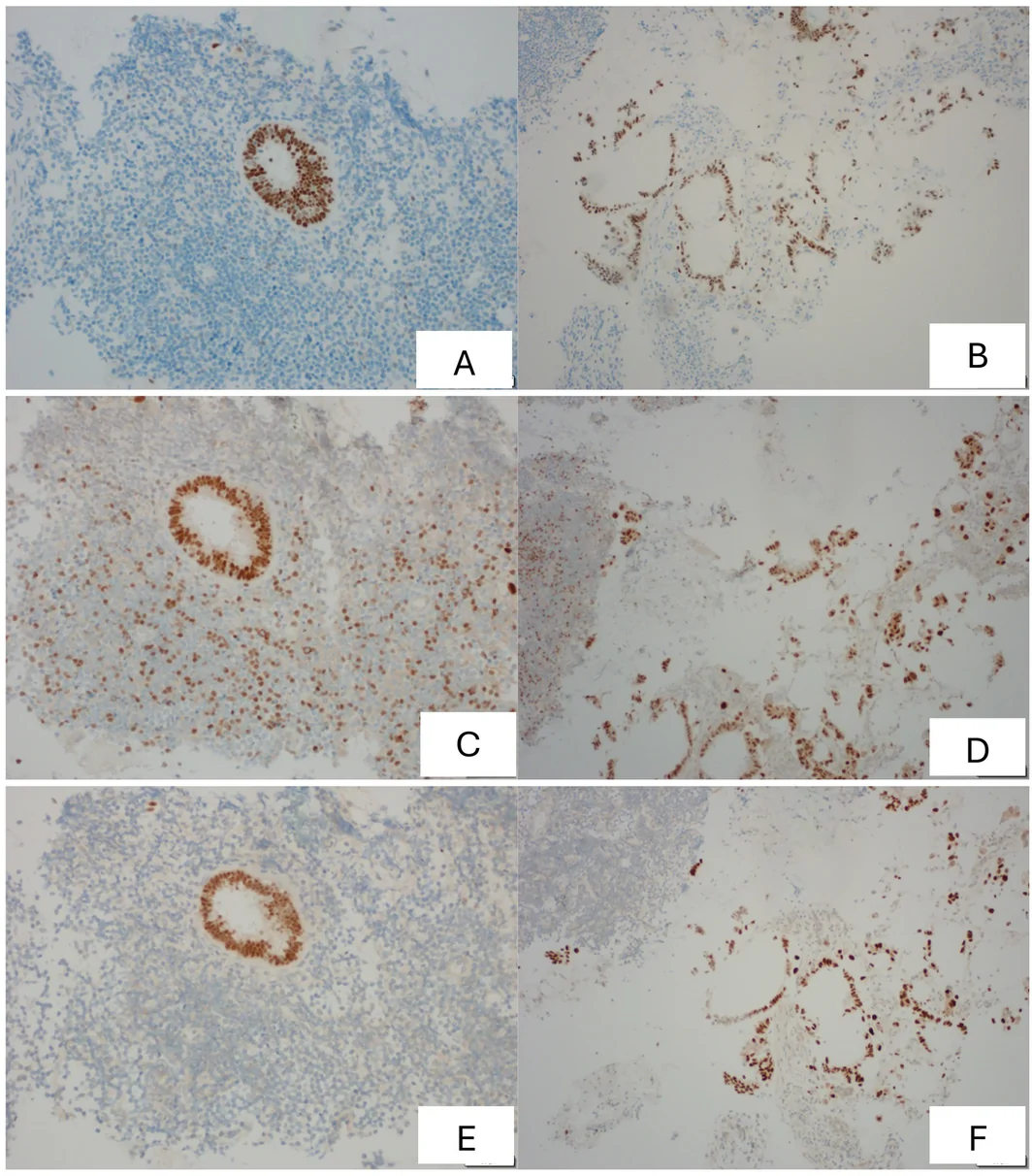
Immunohistochemical stains showing positive nuclear staining for estrogen receptors, PAX‐8, and WT‐1 in endosalpingiosis (A, C, E) and LGSC (B, D, F).

An additional PET/CT scan showed a surgically absent uterus with bilateral pelvic and retroperitoneal surgical clips without associated hypermetabolism. No hypermetabolic foci were identified in the hysterectomy surgical bed, and no significant hypermetabolic lymphadenopathy was identified in the abdomen or pelvis. There was no evidence of peritoneal disease. The carcinoembryonic antigen CEA level was (< 2.0 ng/mL), which was within the normal range.

Review of the pelvic‐washing cell block sections prepared at the time of hysterectomy revealed numerous papillary clusters with focal mild nuclear atypia and psammoma bodies, consistent with involvement by SBT (Figure 4).

**FIGURE 4:**
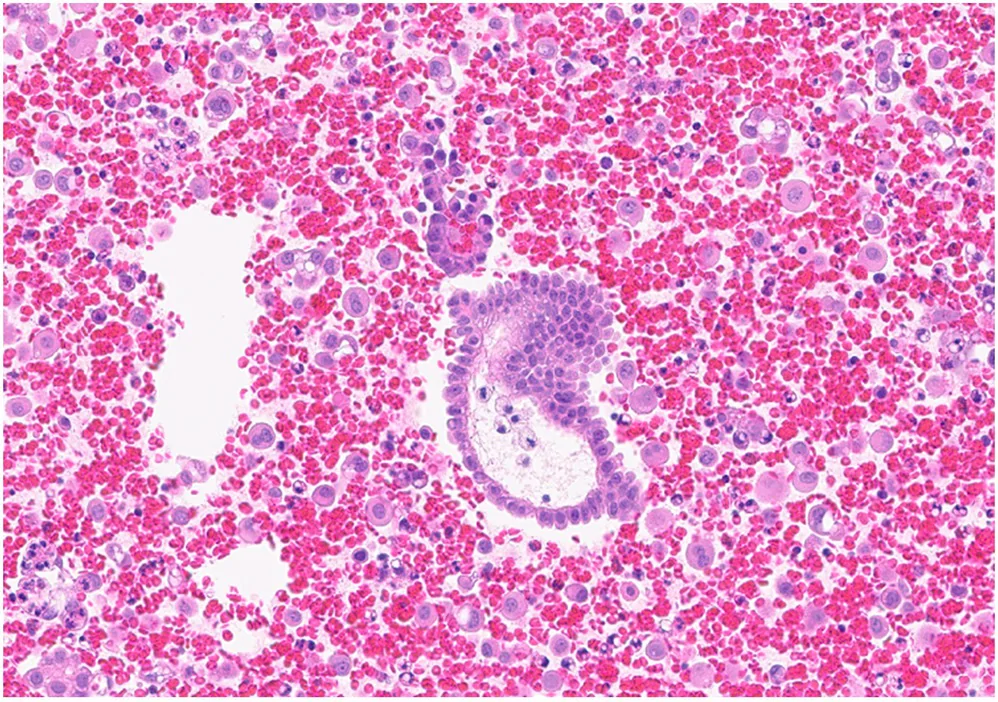
Cell block sections from pelvic washing collected at the time of hysterectomy showing papillary epithelial clusters with minimal atypia, consistent with involvement by a low‐grade serous neoplasm.

The patient's pathology report of the hysterectomy and bilateral salpingo‐oophorectomy, which had been performed 12 years earlier, stated the presence of papillary SBT on both the right ovarian surface (tumor measured 3.5 cm) and left ovarian surface (tumor measured 8.3 cm) and involvement of both fallopian tubes, the posterior cul‐de‐sac, and the bladder peritoneum by noninvasive implants. One of four right pelvic lymph nodes examined was also involved by SBT. Next‐generation sequencing performed on an ovarian specimen showed 2 pathogenic variants: *KRAS* exon 2 c.34G>C (p.G12R) mutation, with a variant allele frequency of 39%, and *MAP2K1* c.157T>A (p.F53I) mutation, with a variant allele frequency of 41%.

Given the patient's history of SBT, the lack of evidence of hypermetabolic lesions in the surgical bed, and the lack of peritoneal disease at the time of diagnosis of the lesion in the left supraclavicular node, the present lesion was favored to be a primary LGSC arising in the setting of nodal endosalpingiosis. The patient underwent neck dissection of the left neck Level IV lymph node and pathology confirmed LGSC in the vicinity of the endosalpingiosis (Figure 5).

**FIGURE 5:**
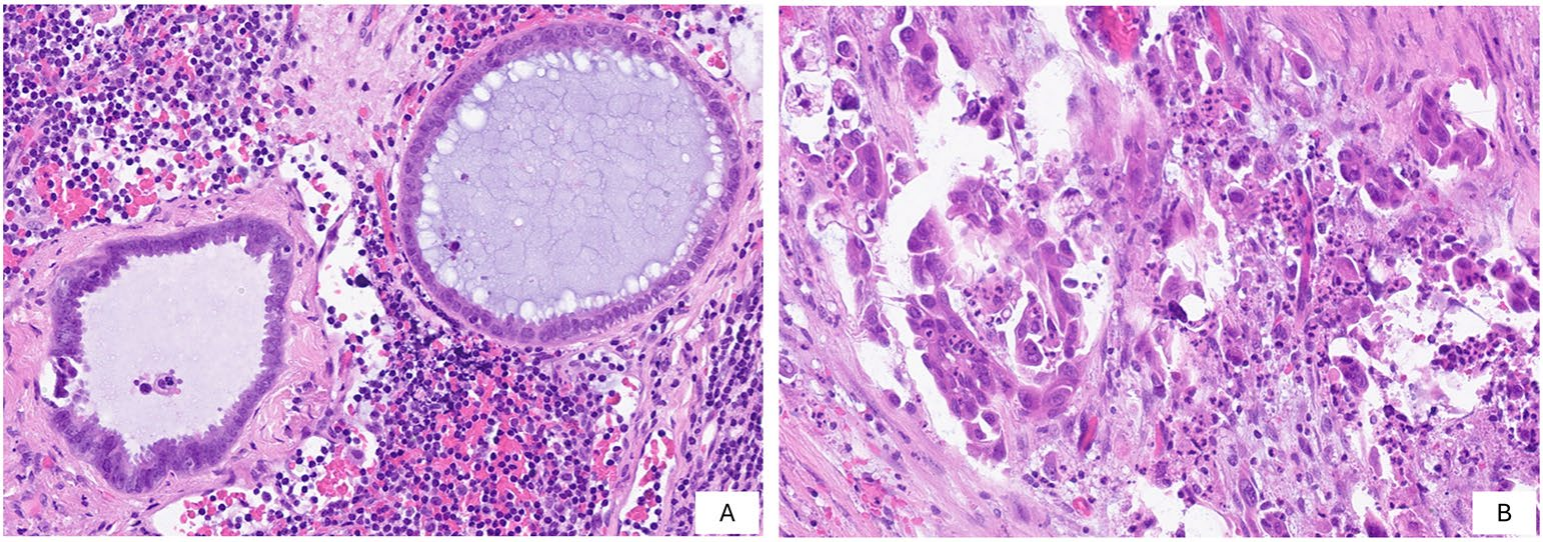
Tissue section of the excised Level IV lymph node showing endosalpingiosis (A) and LGSC (B).

## 3 Discussion

Although endosalpingiosis was initially considered an incidental finding, more recent studies have identified an association between endosalpingiosis and gynecologic malignancy, with 40%–51% of patients diagnosed with endosalpingiosis also having a concurrent gynecologic malignancy [5, 6, 7]. Esselen et al. [5] reported a statistically significant association between endosalpingiosis and uterine and ovarian cancers, particularly SBT, clear cell carcinoma, and mucinous ovarian carcinoma. The rate of nodal endosalpingiosis was also reported to be higher in lymph nodes involved by SBT than in those not involved by SBT (66% vs. 14%) [8].

SBT, which had been diagnosed in our patient 12 years before the presentation described herein, is a noninvasive, low‐grade, proliferative serous epithelial neoplasm. Histologically, typical SBT is characterized by hierarchical branching papillae with variable amount of stroma in the cores. A micropapillary/cribriform pattern is also recognized and is characterized by the presence of elongated micropapillae without stromal cores that directly eminate from large papillae and/or small punched‐out cribriform spaces is [9]. The overall prognosis of SBT is excellent, and the recurrence rate is less than 10%. However, the rate of tumor recurrence has been reported to be higher in cases of SBT associated with endosalpingiosis, and recurrent tumors tended to be LGSC [10].

In contrast with SBT, LGSC is an invasive serous tumor with small nests, glands, papillae or micropapillae, and inverted macro papillae, frequently free‐floating within unlined clear spaces; low‐grade cytological atypia (< 3‐fold variation in nuclear size); and low mitotic activity. Psammoma bodies are often present. Necrosis is rare. These tumors are frequently associated with a coexisting SBT [9].

While serous carcinoma is divided into LGSC and high‐grade serous carcinoma, these entities represent distinct tumor types with a different underlying pathogenesis, not low‐grade and high‐grade variants of the same neoplasm. High‐grade serous carcinomas are associated with *TP53* mutations (more than 97%) and homologous recombination deficiency including *BRCA* mutations, while LGSCs are characterized by *BRAF* or *KRAS* mutations [11, 12].

Several studies have reported cases of nodal endosalpingiosis with subsequent transformation into BST and/or serous carcinoma, sometimes even in the absence of an ovarian primary tumor [8, 13, 14, 15]. Other studies have suggested that the precursor lesion of LGSC is SBT or endosalpingiosis [16, 17]. Somatic mutations in either *KRAS* or *BRAF* were detected at a significantly higher frequency in endosalpingiosis associated with ovarian SBT or LGSC (11/21 [52%]), with most cases (10/11 [91%]) demonstrating identical mutations in both tumors and endosalpingiosis than in endosalpingiosis not associated with ovarian tumors (1/13 [8%]) [16]. These findings support that endosalpingiosis represents an interesting example of the presence of cancer driver mutations in deceptively normal‐appearing cells, which may be prone to neoplastic transformation upon bypass of endogenous oncosuppressive mechanisms [16]. The MAPK pathway appears to play an important role in the pathogenesis of these tumors.

The case reported here underscores several diagnostic considerations: Endosalpingiosis can involve cervical nodes and may progress to SBT and/or LGSC. Although cervical lymph node involvement by endosalpingiosis is extremely rare, awareness of this entity and distinguishing serous neoplasms arising in this setting from metastatic thyroid carcinoma, including papillary thyroid carcinoma, is necessary. Furthermore, in the case we report, as demonstrated by others [10, 17] the long interval between the resection of the SBT and the detection of LGSC and endosalpingiosis in the cervical lymph node suggests that the LGSC in this lymph node most likely represented an independent primary tumor. While a diagnosis of low‐grade serous neoplasm is possible on cytologic findings, the invasive pattern of LGSC can be only visualized on a biopsy/excision specimen.

## 4 Conclusion

This case illustrates a rare instance of LGSC arising within cervical lymph node endosalpingiosis and an exceptionally unusual presentation of Horner syndrome. Recognition of this finding is crucial to avoid mistaken diagnosis of metastasis from an occult primary tumor or recurrent tumor. Thorough clinical, radiologic, and histomorphology evaluation is necessary, and individualized management may be appropriate.

## Author Contributions

**Nyein Nyein Htun:** study conception, data collection, literature search, manuscript preparation/revision. **Nour Sneige:** study conception and design, literature search, interpretation of results and manuscript preparation/revision.

## Funding

The authors have nothing to report.

## Conflicts of Interest

The authors declare no conflicts of interest.

## Data Availability

The data that support the findings of this study are available on request from the corresponding author. The data are not publicly available due to privacy or ethical restrictions.

## References

[1] K. N. Krupa and W. D. Walker , “Endosalpingiosis,” in StatPearls (StatPearls Publishing, 2025). 41802037

[2] A. Cimino‐Mathews , “Axillary Lymph Node Inclusions,” Surgical Pathology Clinics 11, no. 1 (2018): 43–59, 10.1016/j.path.2017.09.004. 29413659

[3] A. H. Salehi , G. Omeroglu , Y. Kanber , and A. Omeroglu , “Endosalpingiosis in Axillary Lymph Nodes Simulating Metastatic Breast Carcinoma: A Potential Diagnostic Pitfall,” International Journal of Surgical Pathology 21, no. 6 (2013): 610–612, 10.1177/1066896913502228. 24021901

[4] E. Davaro , P. Rohra , U. Kundu , and Q. Gan , “Bilateral Cystic Endosalpingiosis in Cervical Lymph Nodes Mimicking Metastatic Papillary Thyroid Carcinoma Morphologically on Cytology: A Case Report and Literature Review of Diagnostic Challenges,” Diagnostic Cytopathology 53, no. 3 (2025): E46–E50, 10.1002/dc.25430. 39679640

[5] K. M. Esselen , K. L. Terry , A. Samuel , et al., “Endosalpingiosis: More Than Just an Incidental Finding at the Time of Gynecologic Surgery?,” Gynecologic Oncology 142, no. 2 (2016): 255–260, 10.1016/j.ygyno.2016.05.036. 27261327

[6] G. K. Lewis , S. Ghaith , E. C. Craver , et al., “The Association of Endosalpingiosis With Gynecologic Malignancy,” Gynecologic Oncology 167, no. 1 (2022): 81–88, 10.1016/j.ygyno.2022.07.025. 35909004

[7] M. Hermens , A. M. van Altena , J. Bulten , A. G. Siebers , and R. L. M. Bekkers , “Increased Association of Ovarian Cancer in Women With Histological Proven Endosalpingiosis,” Cancer Epidemiology 65 (2020): 101700, 10.1016/j.canep.2020.101700. 32146389

[8] B. Djordjevic , S. Clement‐Kruzel , N. E. Atkinson , and A. Malpica , “Nodal Endosalpingiosis in Ovarian Serous Tumors of Low Malignant Potential With Lymph Node Involvement: A Case for a Precursor Lesion,” American Journal of Surgical Pathology 34, no. 10 (2010): 1442–1448, 10.1097/PAS.0b013e3181f17d33. 20871218

[9] International Agency for Research on Cancer. WHO Classification of Tumours Editorial Board , Female Genital Tumours [Internet] (International Agency for Research on Cancer, 2020), https://tumourclassification.iarc.who.int/chapters/34.

[10] E. G. Silva , C. Tornos , Z. Zhuang , M. J. Merino , and D. M. Gershenson , “Tumor Recurrence in Stage I Ovarian Serous Neoplasms of Low Malignant Potential,” International Journal of Gynecological Pathology 17, no. 1 (1998): 1–6, 10.1097/00004347-199801000-00001. 9475184

[11] I. A. Voutsadakis , “Low‐Grade Serous Ovarian Carcinoma: An Evolution Toward Targeted Therapy,” International Journal of Gynecological Cancer 30, no. 10 (2020): 1619–1626, 10.1136/ijgc-2019-000832. 31780569

[12] A. De Leo , D. Santini , C. Ceccarelli , et al., “What Is New on OvarianCarcinoma: Integrated Morphologic and Molecular Analysis Following the New 2020 World Health Organization Classification of Female Genital Tumors,” Diagnostics 11 (2021): 697, 10.3390/diagnostics11040697. PMC8070731 33919741

[13] K. S. Carrick , J. S. Milvenan , and J. Albores‐Saavedra , “Serous Tumor of Low Malignant Potential Arising in Inguinal Endosalpingiosis: Report of a Case,” International Journal of Gynecological Pathology 22, no. 4 (2003): 412–415, 10.1097/01.pgp.0000092155.33490.52. 14501827

[14] S. A. Solaru , M. C. Liu , V. Lee , and R. E. Bristow , “Isolated Low‐Grade Serous Carcinoma Arising in Inguinal Lymph Nodes in the Setting of Endosalpingiosis: A Case Report,” Gynecologic Oncology Reports 59 (2025): 101769, 10.1016/j.gore.2025.101769. PMC12158510 40502817

[15] S. Sah , R. Fulmali , and W. G. McCluggage , “Low‐Grade Serous Carcinoma Arising in Inguinal Nodal Endosalpingiosis: Report of 2 Cases and Literature Review,” International Journal of Gynecological Pathology 39, no. 3 (2020): 273–278, 10.1097/PGP.0000000000000613. 31157685

[16] M. H. Chui and I. M. Shih , “Oncogenic BRAF and KRAS Mutations in Endosalpingiosis,” Journal of Pathology 250, no. 2 (2020): 148–158, 10.1002/path.5353. PMC8328151 31576556

[17] C. L. Ho , R. J. Kurman , R. Dehari , T. L. Wang , and I. M. Shih , “Mutations of BRAF and KRAS Precede the Development of Ovarian Serous Borderline Tumors,” Cancer Research 64, no. 19 (2004): 6915–6918, 10.1158/0008-5472.CAN-04-2067. 15466181

